# The unheard voice: a qualitative exploration of companions' experiences of liaison psychiatry and mental health crises in the emergency department

**DOI:** 10.1192/bjb.2019.2

**Published:** 2019-10

**Authors:** Jennifer Collom, Emma Patterson, Geoff Lawrence-Smith, Derek K. Tracy

**Affiliations:** 1 School of Medicine, St George's University of London, UK; 2 Warwick Medical School, The University of Warwick, UK; 3 Oxleas NHS Foundation Trust, UK; 4 Department of Psychosis Studies, The Institute of Psychiatry, King's College London, UK; 5 Department of Psychiatry, University College London, UK

**Keywords:** Emergency department, carers, companions, qualitative research, liaison psychiatry

## Abstract

**Aims and method:**

To understand the experience of companions of patients seen in the emergency department by liaison psychiatry teams. Participants were recruited via purposive sampling following a recent visit to the emergency department of an inner- or outer-London hospital. Semi-structured interviews were administered to all participants.

**Results:**

Two major themes were generated. The first concerned the appropriateness of the clinical space, in which ‘noise’, ‘privacy’ and the ‘waiting area’ were subthemes. The second was communication with staff, including subthemes of ‘wanting more information’ and a ‘desire to be more involved’.

**Clinical implications:**

Liaison psychiatry services should consider appropriateness of the clinical space, promoting improved communication between staff and patients' companions, and a review of the information provided to companions in the emergency department. This research offers a novel perspective on liaison psychiatry and will enhance current understanding and clinical practice.

## Background

Hospital emergency departments have seen a 50% increase in demand over the past decade and approximately 5% of attendances are for mental health assessment and treatment.^1^ Liaison psychiatry bridges this interface, and for many patients it is their first point of contact with National Health Service (NHS) mental health services. Nationally, there are considerable drivers for continued development of such services, including the Five Year Forward View^2^ and the Crisis Care Concordat.^3^ The government has pledged £247 million to liaison psychiatry services in emergency departments over the next 5 years.^2^

The Care Quality Commission^4^ evaluated patient experience of crisis services, finding considerable dissatisfaction specifically with the quality of liaison psychiatry services. Further, three studies conducted in NHS settings found similar concerns, particularly around waiting times, communication and perceived lack of information, with services described as ‘punitive’.^5^^–^^7^ As an interesting parallel, liaison psychiatry staff reported concern over quality of care caused by ‘poor communication,’ ‘patient dignity not respected’ and ‘delay in investigation or treatment’.^8^ Research has yet to explore these services from a companion's perspective, particularly those who accompany individuals to the emergency department during the acute crisis – individuals who often provide vital emotional support, as well as crucial collateral information to clinicians. Their views may considerably affect individual's attendance at, and engagement with, the emergency department and mental health services more generally.

## Aims

We aimed to explore the perceptions and experiences of companions attending the emergency department with loved ones who were presenting for first-time help-seeking with an acute mental health crisis. This is one part of a two-part study: the other study, which has not yet been published, explored the perceptions and experiences of the patients themselves.

## Method

### Design

This research adopted a qualitative design, most appropriate when aiming to understand the experience of a service from the perspective of the patient rather than hypotheses testing or clinical effectiveness.^9^ The study obtained ethical approval from the Research and Development Department of the Oxleas NHS Foundation Trust.

### Setting

The study took place in one inner- and one outer-London acute general hospital. These are each served by a 24-h liaison psychiatry team with a range of psychiatric, nursing and psychology inputs.

### Participants

Systemic purposive sampling was used to reflect a range in age, gender and ethnicity of companions consistent with that typically seen by the emergency department to maximise the range of experiences captured. We did not try to further stratify based on the presenting problem or diagnosis of their friend or loved one. Participants (*n* = 9) had visited the emergency department between July and August 2015. The sample comprised relatives/friends/companions (collectively referred to as companions) who accompanied a patient following a mental health crisis or mental health crisis–induced physical injury. No payment was offered for participation. There were no drop-outs or data removed following interview (Table 1).

**Table 1:** Participant demographics

Participant no.	Age	Gender
1	52	Female
2	42	Female
3	48	Female
4	48	Female
5	70	Male
6	40	Male
7	52	Female
8	35	Male
9	53	Female

### Materials

A semi-structured interview was designed following an initial review of the literature, and in conversation with senior experienced clinicians. The interview addressed communication; caring and understanding; timeliness; environment; the process of choosing to attend an emergency department and questions regarding the overall experience. We did not try to limit discussion to emergency department staff clearly identified as belonging to the liaison psychiatry service, but encouraged participants to discuss their broader experiences in the emergency department when seeking mental health crisis support. This was because we felt it optimal to capture this wider experience as a truer account of such attendance, and also as participants might not have been certain as to which ‘team’ a given member of staff belonged.

### Procedure

Participants were contacted either in person following assessment in the emergency department, or via telephone to arrange an interview. Written informed consent was obtained following provision of participant information. Interviews were conducted face to face and via telephone (1:8). Interview recordings were transcribed by a researcher.

### Data analysis

Thematic analysis was utilised as it allowed for flexibility and theoretical freedom, and had a descriptive rather than interpretative function.^10^ Primary analysis was undertaken by two researchers (J.C. and E.P.). Important concepts were translated into categorised codes of themes and subthemes that were partly shaped by the interview questions and partly generated from the data. The themes were reviewed with the senior researcher (D.K.T.), and then defined, named and organised into a report with extracts for illustration.

## Results

### Themes

Two over-arching themes were generated: appropriateness of the clinical space and communication with clinical and support staff. As overarching findings, participants felt the environment did not provide enough privacy and was noisy, and communication was considerably below what they would expect or hope for. Participants commented on the lack of information they received and how they felt they could have been more involved in the assessment process. Perhaps unsurprisingly given the wider literature on the topic, nearly every participant commented unfavourably on the waiting time to be seen by the liaison psychiatry team; there was a strong sense of being ‘forgotten’.

#### Appropriateness of the space

It was not felt that the general emergency department space was appropriate for somebody experiencing significant mental distress. Participants noted excessive noise levels and lack of privacy, with assessments variously carried out in ‘a cubicle with a curtain,’ ‘a small office,’ ‘on the Clinical Decisions Unit (CDU) ward’ or ‘a private room’ in the emergency department.

A busy emergency department can be a pressured environment filled with noise and movement, and this was described by one participant as ‘distressing for anybody but particularly for somebody who's in a fairly anxious state’, and a separate area for people with mental health difficulties was commonly suggested. Participants generally felt the emergency department was not appropriate for someone in crisis: one participant noted that it made them feel ‘a little on edge’, and another suggested it could have exacerbated the problem:

‘Because of the specific things that trigger and cause additional, you know, exacerbating like mental health conditions if we could have been in a room, if there was a space in the hospital that wasn't as noisy … you could hear people that were quite distressed … there were people crying out for help and that was really disturbing my sister … it was quite distracting, for her to try and explain how she was feeling and to hear people calling out for help.’ (Participant 3)

The lack of privacy was criticised. One participant described the uncomfortable nature of his daughter being assessed:

‘No, it's not comfortable enough it was in a in the main, a main corridor, people are walking past you just don't feel private enough.’ (Participant 4)

‘Maybe they [people with a mental illness] should be put into a different part not sit in A&E with people that's got broken bones and things … they should have a little bit more privacy.’ (Participant 9)

Another participant commented on her concerns about other patients waiting in the emergency department may negatively view and stigmatise attendees with mental health problems:

‘I've been with my sister and she's been even more agitated than she was, and I think that's probably not well understood by other people waiting in the area.’ (Participant 3)

Interestingly, one companion referred to their relative as ‘different’ to those with physical health problems, adding ‘it's a totally different thing that he went there for’, which resulted in a desire to wait somewhere separate to those with physical injury. Another respondent recommended a separate emergency department entirely for mental health emergencies, stating ‘If there was a mental health A&E, then we'd go there’.

However, some respondents noted that the emergency department could be adequate, understanding that one could not always experience a silent and private space. One stated:

‘Well yes because at the end of the day my wife couldn't really move and that, you know, so yeah yes I mean it would have been nice if it was discussed in a private room, but you know my wife has got trouble with her knees anyway.’ (Participant 6)

#### Communication with staff

Communication with staff is a central part of experiencing a liaison psychiatry service. Some companions were pleased with the communication they received, and felt understood. For example, one participant stated, ‘I felt very much involved and I think they understood my role very clearly and respected that’. However, other companions wanted more information and to be more involved.

Participants commonly expressed a desire for more information regarding patients' care, especially noting their desire for more information to be able to provide appropriate care when they left the emergency department:

‘You know this person's gonna need support, as much support as they can possibly get, but you can only support them to a certain extent if they haven't got the right information.’ (Participant 2)

Others suggested more information specifically on practical steps to undertake during a mental health emergency:

‘There should be some information printed somewhere telling you what to do in a case like this… Or available to close relatives or whatever, I don't know because I didn't know what to do that day.’ (Participant 4)

Some participants noted how they had not been aware of the existence of the liaison psychiatry service. One patient was referred by their general practitioner and their companion stated, ‘I don't see why the GP sent him here, it's for injuries’. Another respondent who had accompanied a patient to the emergency department following an overdose remarked, ‘I didn't expect anyone from mental health to come and see us to be honest … not in A&E’. These comments could demonstrate a lack of awareness of liaison psychiatry; however, they could also suggest previous difficulty in accessing mental healthcare in the emergency department.

Some participants suggested they would have liked to be more involved in the assessment process. For example, one suggested she was not fully aware of the discussed aftercare the patient would receive:

‘Obviously you know last night she wanted to speak with [patient] … it's understandable, but obviously, you know, the person who's with them, or the next of kin should be fully involved with what is actually being said … the aftercare sort of thing.’ (Participant 2)

Another felt unable to be involved with discussions about his daughter because there was no opportunity to do so with sufficient privacy with the liaison staff:

‘You can't talk about your daughter who's got mental health problems when she's in the same room.’ (Participant 4)

On the contrary, some felt that their involvement might have removed the focus away from the patient, which could have been detrimental. For example. one participant stated, ‘If it goes off the person that needs the attention, it may trigger something’.

Several companions expression a sensation of feeling they had been left alone, commenting that they were ‘hanging in the lurch for a long time,’ and ‘you feel as though you've been forgotten’. One participant explained this:

‘With the A&E department it was more, no we wasn't really informed that much, you know, it was kind of, I don't know, was just kind of left there, waiting a long time.’ (Participant 1)

Generally people expected a long wait and understood this; for example, one participant stated ‘I literally knew the minute we left I knew once we're going to A&E we're gonna have a wait so that wasn't unexpected’, and another said ‘I can understand the waiting time’. However, it appears that during this wait, more could have been done to keep people informed of progress or delays:

‘If they, the waiting staff were made more informed with what's actually happening with the patients in front of you, or whatever, that would be helpful.’ (Participant 8)

‘it's just information; you just feel so, “what the hell is going on”?’ (Participant 8)

‘It would have been nice to have said… “We haven't forgotten you.”’ (Participant 1)

## Discussion

This study aimed to better understand the experience of the liaison psychiatry service from a companion's perspective – the first time, to the best of our knowledge, that this has been undertaken – to assist reflection and improvement of care provision. Through qualitative interviews, two main themes were generated from the data: appropriateness of the space and communication with staff.

The qualitative literature on experiences of liaison psychiatry is sparse, and inconsistent in the quality of methodology, with recent calls for more such research in this field.^11^ This supports the objectives of the Crisis Care Concordat^3^ that called for research that shared the views of patients, and companions of patients, of mental health crisis services. Further, Parsonage *et al*^12^ suggested that there is a need for evaluative rather than descriptive research, which measures liaison psychiatry from a perspective other than improvement in mental health.

Successive governments have expressed a desire to increase parity between mental and physical health by, at least in part, enhancing and developing liaison psychiatry services.^13^^,^^14^ However, companions regularly inferred that the emergency department was not a suitable place, as mental health is ‘different’ to physical emergency. This is a complex debate, and there are counterarguments that a ‘psychiatric emergency department’ could perpetuate stigma, as well as potentially increasing risks due to putative problems in accessing any necessary physical health checks or care. There is a growth, albeit sporadic, of ‘crisis cafés’ to augment out-of-hours emergency mental health services such as liaison psychiatry, and crisis and home treatment teams. These will likely offer patients an alternative to the emergency department, although their effectiveness has yet to be properly evaluated on a large scale.

The findings from this research could be used to facilitate the development of routine outcome measures, distributed to companions and patients in hospitals nationwide. This is in line with the NHS quality agenda of enhancing patient experience.^15^ Quantitative tools necessary for wide scale service evaluation must be based on qualitative research such as this.

Although the experiences of companions attending the emergency department with an individual during mental health crisis have not been researched qualitatively, patient and staff experiences have been studied, and support the current findings. Eales *et al*^5^ found participants were more likely to leave before being seen if there was a longer wait time, whereas general emergency department staff reportedly viewed mental illness as ‘low status’ and were hostile toward suicidal patients.^16^^,^^17^ This could provide an explanation for the reduced communication from emergency department staff toward companions in this study. The environment and communication have been found to be the area of liaison psychiatry most in need of attention.^18^ Noblett *et al* reported that staff had concerns over quality of care caused by ‘poor communication,’ ‘patient dignity not respected’ and ‘delay in investigation or treatment’.^8^ It is apparent that staff, patients and now those who accompany patients all feel these are areas requiring improvement and must not be ignored.

A report published by the National Confidential Enquiry into Patient Outcome and Death^19^ looked at mental health treatment in the general hospital setting, finding that compared with visiting hospital with a physical health condition, less information was given to relatives and carers. The authors recommended that all general hospital staff who have interaction with patients, including clinical, clerical and security staff, should receive training in mental health conditions. Adequate communication with patients, their family and carers was said to be essential for good care, but this was considered as inadequate in 35.3% of cases. This report supports the finding of this study, in that more information should be given to companions and communication between hospital staff and patients' needs to improve.

### Novelty of this research

Government reports and much of the literature suggest companions and relatives should be more involved in research into services and decisions regarding patient care.^3^^,^^4^ Yet, this cohort is significantly underrepresented within mental health research, and has not been included in any qualitative assessment of liaison psychiatry services. Research has previously addressed patient and staff experiences, and has found similar themes; however, the research is dated and mostly quantitative. Adding the experiences of companions to the literature is both necessary and valuable to best improve liaison psychiatry with the interest of its patients in mind. This study is, to the best of our knowledge, the first of its kind, in the UK and elsewhere.

### Limitations

Qualitative research methods enable detailed information about liaison psychiatry services from a companion's perspective, which would not have been obtainable via quantitative methods. Adopting this technique, however, limits the number of participants from whom data can be obtained; this and the fact that the study covered two sites in South-East London limit the generalisability of the research. More participants might have been interviewed, and further novel data obtained, although we felt that there was reasonable saturation in the data being obtained through the last few interviewed. Participants' data might have been affected by reciprocity if they felt an obligation to refer to the liaison psychiatry service favourably. Yet, detailed and uniquely sighted data such as that collected here would not be accessible via quantitative methods. Further, the study was limited by employing a single researcher to conduct the interviews and analyse the data. We intentionally set out to explore the overarching experience of being in the emergency department when seeking help for a mental health crisis. This very commonly involves interactions with staff from different departments, from emergency department staff to other specialties such as cardiology and so forth. Some participant experiences may thus be based around ‘non-mental health staff’ who may be less versed or comfortable with managing individual with mental health problems. Nevertheless we felt it preferable to try capture this wider experience, not least as members of the public may be unsure as to which ‘team’ or service a given staff member belonged.. We did not identify any common themes based on the types of presentation of loved ones, although the small numbers involved and the nature of qualitative research meant that we did not set out to find such. Finally, the research would have benefitted from patient and public involvement in the interview design process.

### Clinical implications

The intention of this research was to understand the liaison psychiatry service from a companion's perspective, with an aim to improve services and inform the development of future services. Liaison psychiatry is critical in establishing a connection between mental and physical health, and its value is recognised by the government. Following this research, some changes to liaison psychiatry services should be considered.

First, the environment in which patients and companions wait to be seen should reviewed, with an aim to introduce quieter and calmer areas where necessary. Second, the degree of involvement of companions in assessments should be agreed upon collaboratively with the patient, to utilise their (often) expert knowledge of the patient. Finally, companions and patients require more information about the liaison psychiatry service. Individuals already known to services should be made aware of the emergency department as an appropriate and welcome place to go during a crisis. When people are waiting to be seen, information should be provided explaining why they have been referred to liaison psychiatry and what this means for their care. This information should be supported by improved communication between emergency department staff and patients during waiting.

The need to implement these changes is further supported by a parallel study by our team (Patterson *et al*, in preparation), which looked at patient experience of liaison psychiatry and the emergency department. There were three areas of clear overlap: (a) patients also desired clear and compassionate communication from staff, (b) patients suggested that more awareness of liaison psychiatry was essential and (c) they felt that the waiting and clinical assessment areas lacked privacy and were noisy. The areas that were not common between this paper and the current study were unique to either the patient or companion experience. For example, companions wishing to be more involved, and patients reporting ‘mental overload’. The similarities in concerns between companions and patients supports the need to address these areas of liaison psychiatry and the emergency department in general for people experiencing mental health crisis.

### Future research

The current study should be replicated with a more nationally representative sample and a larger number of services. An additional direction for future research is to discover ways of reducing the number of patients leaving the emergency department without treatment. Additional qualitative research should aim to gather staff's views on the proposed improvements from companions and patients as well as enquiring about their own. To summarise, liaison psychiatry is an essential service within the general hospital, providing help to people with coexisting mental and physical health complications, and those in crisis with nowhere else to turn. Research has suggested improvements need to be made to this service as its patients do not report consistent satisfaction.^4^ This study proposes improvements must be made to the environment and the communication between staff and patients/companions, and more information should be made available. Further, this research has offered companions the opportunity to share their experience, a worryingly neglected cohort, able to offer valuable information to aid service improvement.

## About the authors

**Jennifer Collom** is a medical student at St George's University of London, UK. **Emma Patterson** is a medical student at Warwick Medical School, University of Warwick, UK. **Geoff Lawrence-Smith** is a consultant liaison psychiatrist with the Oxleas NHS Foundation Trust, UK. **Derek K. Tracy** is a consultant psychiatrist and Clinical Director at Oxleas NHS Foundation Trust; a visiting senior lecturer with the Department of Psychosis Studies, Institute of Psychiatry, King's College London; and a visiting senior lecturer with the Department of Psychiatry, University College London, UK.
